# Recent advances of trace elements in autoimmune thyroid disease

**DOI:** 10.3389/fimmu.2025.1662521

**Published:** 2025-11-26

**Authors:** Shanshan Li, Qian Xu, Shengjun Wang, Huiyong Peng, Yingzhao Liu

**Affiliations:** 1 Department of Endocrinology, The Affiliated People’s Hospital of Jiangsu University, Zhenjiang, China; 2 Department of Laboratory Medicine, The Affiliated Hospital of Jiangsu University, Zhenjiang, China; 3 Department of Laboratory Medicine, The Affiliated People’s Hospital of Jiangsu University, Zhenjiang, China

**Keywords:** trace elements, micronutrients, immune regulation, autoimmune thyroid disease, hormones

## Abstract

Autoimmune thyroid disease (AITD), which includes Graves’ disease and Hashimoto’s thyroiditis, poses a substantial global health burden due to its strong environmental influences. Among environmental factors, trace elements are increasingly recognized for their dual roles in regulating thyroid physiology and immune function. This narrative review synthesizes current evidence on the roles of key trace elements-iodine, selenium, vitamin D, zinc, iron, copper, and magnesium-in AITD pathogenesis. We detail their essential functions in thyroid hormone synthesis and metabolism, and critically examine how imbalances (deficiency or excess) can disrupt immune homeostasis, thereby promoting autoimmunity via mechanisms like oxidative stress, aberrant immune cell differentiation, and loss of self-tolerance. This review highlights complex dose-response relationships, such as the U-shaped curve for iodine, and the protective roles of selenium and vitamin D through antioxidant and immunomodulatory pathways. For other elements, including zinc, iron, copper, and magnesium, emerging associations with AITD have been identified, but the mechanistic understanding remains limited. We conclude that imbalances in trace elements are pivotal environmental triggers for AITD. Future research should prioritize elucidating molecular mechanisms, investigating interactions among elements, and conducting long-term interventional studies to translate these findings into precise nutritional strategies for AITD prevention and management.

## Introduction

Autoimmune thyroid disease (AITD) is an organ-specific disease of the thyroid gland characterized by immune-mediated thyroid dysfunction, thyroid autoantibody production, and lymphocytic infiltration (1). AITD affects 1-5% of the global population, with a 5-10-fold higher incidence in female. In China, the prevalence of hypothyroidism is about 1.02%, whereas subclinical hypothyroidism affects 12.93%, with autoimmune etiologies accounting for > 60% of cases (2). The most common types of AITD include Graves’ disease (GD) (3) and Hashimoto’s thyroiditis (HT), sharing histopathological features of lymphocytic infiltration (4). While genetic predisposition (e.g., HLA-DR and CTLA-4 polymorphisms) accounts for 50-60% of disease susceptibility (5), environmental triggers-particularly trace elements-have garnered increasing attention due to their dual roles in thyroid physiology and immune regulation (6, 7).

Despite minimal bodily concentrations, trace elements exert critical physiological influences (8). Dietary sources often prove insufficient to maintain optimal levels, predisposing individuals to deficiencies (9). Imbalances in these micronutrients can disrupt systemic functions and contribute to disease pathogenesis. Notably, certain trace elements modulate immune responses, cellular proliferation, and tissue repair (10, 11). Their impact on thyroid homeostasis is multifaceted: they regulate hormone synthesis, metabolism, and immune activity, with dysregulation linked to thyrotoxicosis, hypothyroidism, AITD (Graves’ disease and Hashimoto’s thyroiditis), and thyroid malignancies (12). Given the high prevalence and chronic nature of AITD-which imposes substantial socioeconomic burdens on healthcare systems and compromises patients’ quality of life-integrating trace element assessment into clinical practice offers a cost-effective approach for risk stratification and personalized management. This narrative review synthesizes current evidence regarding the roles of key trace elements (iodine, selenium, vitamin D, zinc, iron, copper, and magnesium) in AITD pathogenesis and discusses their potential implications for clinical management and future research.

## The role of trace elements in AITD

### Iodine and AITD

As an essential trace nutrient for the human body, iodine plays a crucial role in growth and development, metabolic regulation, and neurocognitive function. Iodine is an essential raw material for synthesizing thyroid hormones (bioactive triiodothyronine T3, thyroxine T4). Within the thyroid follicular lumen, it combines with tyrosine residues on thyroglobulin under the catalysis of thyroid peroxidase (TPO), ultimately converting into biologically active T3 and T4 (13–15). Since Baumann first discovered the phenomenon of iodine accumulation in the thyroid gland (16), numerous epidemiological studies have shown that iodine intake is associated with a U-shaped curve relationship with the risk of thyroid disease (17). Notably, in iodine-sufficient regions, Hashimoto’s thyroiditis has become the primary cause of hypothyroidism (18), while in high-iodine intake areas, the incidence of AITD significantly increases, particularly with marked elevations in thyroid peroxidase antibodies (TPOAb) and thyroid globulin antibodies (TgAb) levels (19–21).

While these epidemiological observations establish a clear association between iodine exposure and AITD risk, a deeper mechanistic understanding is required to elucidate the causal pathways involved. Mechanistic studies have elucidated the process by which excessive iodine intake disrupts thyroid homeostasis and triggers autoimmunity. At the cellular level, excessive iodine directly inhibits thyroid peroxidase (TPO) activity and sodium-iodide symporter (NIS) expression, thereby disrupting thyroid hormone synthesis (20). It also induces reactive oxygen species (ROS) generation in thyroid epithelial cells, leading to oxidative damage, suppression of protective autophagy, and ultimately, cellular apoptosis (22, 23). These cellular injuries are hypothesized to expose neo-antigens, thereby initiating an immune response. Supporting evidence from animal models of experimental autoimmune thyroiditis shows that high iodine intake amplifies the autoimmune cascade. Specifically, it promotes pro-inflammatory T helper 17 (Th17) cell proliferation, alters peripheral lymphocyte function, and disrupts systemic antioxidant balance and cytokine profiles (24, 25). Collectively, evidence from *in vitro* and *in vivo* studies delineates a coherent pathway from initial cellular damage and thyroid dysfunction to the breakdown of immune tolerance.

These findings reveal a complex dose-response relationship between iodine intake and the development of AITD. While adequate iodine intake is crucial for maintaining thyroid function, excessive intake may increase the risk of AITD through mechanisms such as oxidative stress and abnormal immune regulation. A deeper understanding of these mechanisms not only provides new insights into the etiology of AITD but also offers potential targets for the development of clinical prevention and treatment strategies.

### Selenium and AITD

In 1817, Swedish chemist J.J. Berzelius discovered the element selenium—one of the essential trace elements in the human body (26). Selenium serves as a fundamental element for the normal functioning of human and animal organisms. It accumulates in the form of inorganic selenate compounds (IV) or (VI), which are then converted into organic forms to synthesize selenoproteins (27). These selenoproteins participate in various physiological, biochemical, and metabolic processes within human tissue cells, exhibiting antioxidant, anti-inflammatory, and immune-regulatory functions (28). Current research has identified 25 human-encoded selenoproteins, with the most closely associated with thyroid diseases being the glutathione peroxidase (GPx) family, thioredoxin reductase (TrxR), and iodothyronine deiodinase (DIO) (29).

Selenoproteins play a multifaceted role in the development of thyroid diseases (30–32). Firstly, selenoproteins such as GPx can remove hydrogen peroxide (H_2_O_2_) from thyroid cells, thereby protecting cell membranes. When selenium is deficient in the body, GPx function is inhibited, leading to reduced antioxidant stress resistance in thyroid cells and increased cellular damage and apoptosis. This damage may impair thyroid hormone synthesis. Second, selenium is a core component of selenoproteins and a key regulator of thyroid hormone metabolism. It facilitates the conversion of T4 to T3 through deiodinases (DIO1/DIO2) (33–36). Consequently, selenium deficiency lowers deiodinase activity, which impairs T4-to-T3 conversion, leads to aberrant thyroid hormone levels, and disrupts normal physiological functions. Additionally, thioredoxin in selenium proteins is an important component of cysteine residues in nuclear transcription factors, responsible for regulating thyroid cell differentiation and proliferation. Finally, selenium proteins also possess immune regulatory functions, participating in the activation of T cell proliferation, differentiation, and redox metabolism. Furthermore, selenium is involved in reducing excessive immune responses and chronic inflammation (37). Mice on a high-selenium diet not only increased T Cell Receptor (TCR) signal transduction in CD4^+^ T cells but also increased IL-2 expression, shifting the T Helper 1 Cell/T Helper 2 Cell (Th1/Th2 cell) balance toward the Th1 phenotype, with higher levels of interferon-γ (IFN-γ) and CD40 ligand (38), thereby inhibiting immune damage to thyroid cells and reducing the incidence of AITD. Therefore, it can be concluded that selenium plays a crucial regulatory role in the synthesis, conversion, metabolism, and associated diseases of thyroid hormones.

Selenium is both an antioxidant and an immune stabilizer, it can protect thyroid cells from oxidative stress damage, regulate immune function, and prevent thyroid tissue from being attacked by the immune system (39).In animal studies, selenium supplementation for eight weeks in a high-iodine diet-induced experimental autoimmune thyroiditis (EAT) mouse model significantly attenuated thyroid follicular destruction and lymphocyte infiltration, and improved thyroid hormone and autoantibody levels (40). These animal data provide strong evidence for selenium’s immunomodulatory effects. However, extrapolation to human disease requires caution due to differences in immune responses, disease progression, and selenium metabolism between mice and humans.

In human cross-sectional studies (as shown as **Table 1**), serum selenium levels are negatively correlated with free T4 (FT4) and TSH in GD patients without GO. Similarly, in GD patients with GO, serum selenium levels were negatively correlated with FT3, FT4, and TSH (41). Studies across regions with varying selenium intake further support this, demonstrating that GD patients exhibit lower selenium levels, which correlate with GO clinical manifestations. Lower selenium levels are associated with increased GO risk, and were identified as an independent risk factor for moderate-to-severe GO (42, 43). Moreover, the prevalence of autoimmune thyroiditis (AIT) was higher in selenium-deficient areas than in selenium-adequate areas, suggesting that low selenium is a potential risk factor for AIT (44). In an iodine-sufficient region, serum selenium levels were significantly lower in newly diagnosed GD (97.68 μg/l) and HT (104.36 μg/l) patients compared to healthy controls (122.63 μg/l, p < 0.001) (45). However, observational studies can only indicate associations rather than establish causality, and are susceptible to confounding by factors such as other nutrient intakes, environmental exposures, or genetic background.

**Table 1:** Summary of selected clinical studies on trace elements supplementation in autoimmune thyroid diseases (AITD).

Study (year)	Design	Population	Intervention & duration	Key findings & quantitative data
Owji et al. (2022) (41)	Case-Control	174participants (Iran)	–	Serum selenium was significantly lower in Graves’ disease patients with ophthalmopathy (96.82 ± 30.3 μg/dL) compared to those without (94.53 ± 25.36 μg/dL、) and healthy controls (102.55 ± 16.53 μg/dL).
Kim et al. (2022) (42)	Retrospective Case-Control	91 participants (Korea)	–	Serum selenium levels were significantly lower in Graves patients with or without GO, compared to non-Graves control participants. But Selenium levels were not associated with clinical activity scores or NOSPECS scores.
Liu et al. (2018) (43)	Case-Control	244 participants (China)	–	Serum selenium (Se) levels in EUGD group (median: 7.53 *µ*g/dL), HyGD group (median: 6.76 *µ*g/dL), and GO group (median: 7.40 *µ*g/dL) were significantly lower than those in NC group (median: 9.20 *µ*g/dL, all *P* < 0.01). Hyperthyroidism and thyroid-specific antibodies grade were associated with high Cu levels.
Wu et al. (2015) (44)	Cross-Sectional	6152 participants (China)	–	A lower population selenium status was associated with a higher prevalence of thyroid disease (18.0% vs 30.5%;P <.001), including hypothyroidism, hyperthyroidism, and autoimmune thyroiditis and enlarged thyroid.
Heidari & Sheikhi (2022) (45)	Case-Control	180 participants (Iran)	–	The Se level in patients with HT (104.36 μg/l) and GD (97.68 μg/l) was significantly lower than in the control group (122.63 μg/l) (*P* < 0.001). The incidence of Se deficiency in patients with HT, GD, and in the control group was 15.2%, 2.5%, and 2.5%, respectively (*P* < 0.001).
Nordio & Basciani (2017) (46)	Intervention Study	68 patients with Hashimoto’s thyroiditis (Italy)	Group 1: MI-Se Group 2: Se only Duration: 6 months	TSH, anti-thyroid peroxidase (TPOAb) and anti-thyroglobulin (TgAb) levels were significantly decreased in patients treated with combined MI-Se after six months of treatment. Also, a significant free serum T4 increase was observed in MI-Se group, along with an amelioration of patients’ quality of life.
Pace et al. (2020) (47)	Observational Study	–	Group 1: untreated Group 2: Se-meth: 83 μg/day Group3: Se-meth + Myo-I: 83 μg/day + 600 mg/day Duration: 6–12 months	Compared to baseline, levels of thyroid-stimulating hormone (TSH) increased significantly in untreated patients but decreased by 31% and 38%.
Pirola et al. (2016) (48)	Before-After Study	196 patients with Hashimoto’s and SCH (Italy)	Selenium supplementation (83 mcg/day as selenomethionine). Duration: 4months	17.2% of patients restored euthyroid function (normal TSH) after supplementation.
Zuo et al. (2021) (49)	Meta-analysis	–	–	Meta-analysis results showed that the serum free triiodothyronine (FT3) levels in patients was greatly reduced after selenium supplementation compared to placebo treatment (MD =-0.40; 95% confidential interval (CI): -0.70–0.10; Z = 2.61; P = 0.009). Serum free thyroxine (FT4) levels and anti-thyroid peroxidase antibody (TPOAb) levels were also significantly reduced (MD = -0.76; 95% CI: -1.58–0.07; Z = 1.79; P = 0.07), and anti-thyroid peroxidase antibody (TPOAb) level was decreased observably (MD =-150.25; 95% CI: -04.06–96.43; Z = 5.47; P<0.00001).
Filipova et al. (2023) (67)	prospective case-control study	98 patients	–	No significant association found. The prevalence of vitamin D insufficiency was 60.31% in AITD women and 52.5% in the control group.
Chao et al. (2020) (68)	Cross-Sectional	75436 patients	–	Patients with HT present with a reduced 25(OH)D level, and TSH is an independent risk factor for HT. TSH is negatively correlated with 25(OH)D level. FT3 and FT4 levels were positively correlated with 25(OH)D levels.
Wang et al. (2015) (69)	Meta-analysis	–	–	Compared to controls, AITD patients had lower levels of 25(OH)D (SMD: −0.99, 95% CI: −1.31, −0.66) and were more likely to be deficient in 25(OH)D (OR 2.99, 95% CI: 1.88, 4.74).
Kivity et al. (2011) (70)	observational study	50 patients with AITDs, 42 patients with non-AITDs and 98 healthy subjects	–	The prevalence of vitamin D deficiency was significantly higher in patients with AITDs compared with healthy individuals (72% versus 30.6%; P<0.001), as well as in patients with Hashimoto’s thyroiditis compared to patients with non-AITDs (79% versus 52%; P<0.05). Vitamin D deficiency also correlated to the presence of antithyroid antibodies (P = 0.01) and abnormal thyroid function tests (P = 0.059).
Ertek et al. (2010) (83)	observational study	332 patients		In patients with normal thyroid, zinc levels were significantly positively correlated with free T3 levels (p<0.001). In the nodular goitre group, thyroid volume was negatively correlated with TSH and circulating zinc levels (p=0.014 and p=0.045, respectively). In the AITD group, thyroid autoantibodies and zinc were significantly positively correlated.
Szczepanik et al. (2021) (84)	Case-Control	42 women with Hashimoto’s disease and a control group of 30 healthy women.	–	Compared to the healthy control group (86.5 ± 76.0-104.0), serum zinc levels in women with Hashimoto’s thyroiditis (88.0 ± 82.0-95.0) did not differ significantly,p=0.7217.
Vargas-Uricoechea et al. (2024) (85)	Case-Control	1048 participants	–	The prevalence of low zinc levels in those with hypothyroidism (both subclinical and overt) was 49.1% [odds ratio (OR) of low zinc levels: 5.926; 95% CI: 3.756-9.351]. The prevalence of low zinc levels in participants with hyperthyroidism (both subclinical and overt) was 37.5% [OR of low zinc levels: 3.683; 95% CI: 1.628-8.33].
Parsaei et al. (2025) (88)	Meta-analysis	47studies involving 53,152 pregnant women	–	Iron deficiency was associated with higher thyroid-stimulating hormone (2.31 mIU/L vs. 1.75 mIU/L) and lower free T4 (10.7 pmol/L vs. 13.3 pmol/L).
Wang et al. (2022) (89)	cross-sectional study	2218 pregnant women	–	Compared with normal group, free triiodothyronine (FT3) and free thyroxine (FT4) in iron deficiency group and iron deficiency anemia group had a significant decreasing trend (P < 0.05), with the lowest levels in iron deficiency anemia group.
Zimmermann et al. (2003) (90)	RCT	377schoolchildren (Morocco)	Dual-fortified salt (Iodine + Iron) vs. Iodine-only salt. Duration: 9 months	Dual fortification effectively improved iron and iodine status without inhibiting thyroid function (no significant TSH difference between groups).
Kim et al. (2020) (95)	Retrospective Cohort Study	448 participants (Korea)	–	Both copper and selenium were positively associated with FT4 levels (p<0.001), suggesting a role in maintaining normal thyroid function.
Rasic-Milutinovic et al. (2017) (96)	cross-sectional,Case-Control	22 female patients with Hashimoto thyroiditis vs 55 female healthy persons	–	The ratio of Copper, and Selenium may influence directly thyroid function in patients with HT and overt hypothyroidism.
Mehl et al. (2020) (97)	Cross-Sectional	523 participants	–	The patients displayed relatively low serum zinc and selenium concentrations as compared to a set of healthy subjects (zinc; 1025+/-233 vs. 1068+/-230 μg/L, p < 0.01, selenium; 76.9+/18.8 vs. 85.1+/-17.4 μg/L, p < 0.0001), but Serum copper was not different between the groups.
Wang et al. (2018) (99)	Cross-Sectional	1257 participants (China)	–	Severely low serum magnesium was associated with increased risks of positive TgAb (p < 0.01, odds ratios [ORs] = 2.748-3.236) and hypothyroidism (p < 0.01, ORs = 4.482-4.971).

AITD, Autoimmune Thyroid Disease; SCH, Subclinical Hypothyroidism; TPOAb, Thyroid Peroxidase Antibodies; TSH, Thyroid-Stimulating Hormone; FT3/FT4, Free Triiodothyronine/Thyroxine; OR, Odds Ratio; CI, Confidence Interval; SMD, Standardized Mean Difference; CAS, Clinical Activity Score. RCT, Randomized Controlled Trial. Patients with newly diagnosed Graves’ disease (HyGD), GD patients with euthyroid status or subclinical thyroidism after treatment (EUGD), GO patients with euthyroid status or subclinical thyroidism after treatment (GO), and normal controls (NC).

Based on these associations, several selenium supplementation trials have been conducted. After six months of combined myo-inositol and selenium supplementation, the six-month combined MI-Se regimen significantly reduced serum levels of thyroid-stimulating hormone, anti-thyroid peroxidase antibodies, and anti-thyroglobulin antibodies, indicating reduced antibody titers and prevention of thyroid function deterioration (46, 47).In another trial,4 months of selenium monotherapy (sodium selenite, 83 μg/day) normalized TSH levels in 17.2% (33/192) of subjects. Responders were significantly more frequent among Cases than Controls (30/96 [31.3%] vs. 3/96 [3.1%],resulting in a highly significant intergroup difference (p < 0.0001 (48). Additionally, clinical trials demonstrated that selenium supplementation significantly reduced FT3, FT4, and TPOAb levels in AITD patients (49).

Immunologically, T-cell subsets are key factors in AITD pathogenesis. Studies in autoimmune thyroid models show reduced CD4+CD25+Foxp3+ cell numbers and decreased Foxp3 mRNA expression in splenocytes. Selenium supplementation increases regulatory T cell (Treg) numbers (40, 50). CD4+CD25+Foxp3+ Tregs are essential immune components that suppress autoimmune responses; their deficiency promotes self-reactive immunity and tissue damage (51). Thus, selenium may inhibit antibody production by upregulating Tregs. Selenium supplementation increases Treg proportion and function, suppresses pro-inflammatory cytokine secretion, and delays thyroid follicular cell apoptosis (52). Selenium may regulate immune function in AITD by modulating Th1/Th2 cytokine expression. Th1 cytokines activate macrophages, stimulate complement and antibody production, and exert cytotoxic effects that promote thyroid disease progression. In contrast, Th2 cytokines inhibit Th1 cytokine production (53, 54). Th17 lymphocytes, which share pro-inflammatory properties with Th1 cells, have also been recently implicated (55). In early-stage HT, Th17-dependent responses predominate, whereas Th1 activity increases in later stages (56). A study showed that selenium supplementation modulates interferon-γ and interleukin-1β levels, suggesting it regulates the Th1/Th2/Th17-Treg balance (54). These studies provide evidence for selenium’s immunomodulatory potential in AITD treatment. However, most clinical trials are limited by small sample sizes, short follow-up, and inconsistent selenium forms and dosages, restricting result generalizability and comparability. Furthermore, variations in disease stage and baseline selenium status across studies may confound efficacy assessments.

In summary, evidence from *in vitro*, animal, and human studies progressively constructs a framework for selenium’s protective role in AITD, particularly through regulating T-cell subset balance. However, human studies, particularly intervention trials, have methodological limitations. Future research requires larger, longer-term, rigorously designed randomized controlled trials to determine the optimal timing and target populations for selenium supplementation in AITD prevention and treatment. Furthermore, elucidating the molecular mechanisms of selenium-mediated immune cell differentiation will provide a foundation for its application in AITD precision medicine.

### Vitamin D and AITD

Vitamin D is a fat-soluble vitamin and a derivative of steroids, with multiple biological forms. The two most important fat-soluble steroids for humans are vitamin D2 and vitamin D3 (57). It can be naturally synthesized through sun exposure or obtained through diet, and is converted in the liver into 25-hydroxyvitamin D/25(OH)D, and in the kidneys into 1,25-dihydroxyvitamin D/1,25(OH)_2_D/calcitriol (58). 1,25(OH)_2_D is the primary bioactive metabolite, i.e., the hormonal form of vitamin D, whose primary function is to maintain calcium and phosphorus homeostasis and regulate bone metabolism (59). Furthermore, by binding to the vitamin D receptor (VDR), it modulates the expression of thyroid-related genes and is significantly inversely correlated with TSH levels (60–62). VDR is widely expressed in cells and organs such as vascular endothelial cells, immune cells, and endocrine glands. Notably, immune cells can also synthesize 1α-hydroxylase, which is associated with vitamin D activation. 25-hydroxy vitamin D [25-hydroxy vitamin D, 25-(OH)D] is converted into biologically active 1,25-(OH)2D by hydroxylase and exerts its biological functions through autocrine or paracrine mechanisms. This confirms the role of vitamin D as an immunomodulator in the development of various autoimmune diseases (63). Current research also confirms that vitamin D deficiency is associated with the onset of various autoimmune diseases, including rheumatoid arthritis, systemic lupus erythematosus, multiple sclerosis, and type 1 diabetes (64). Recent domestic and international studies have found that vitamin D also plays a role in diabetes, the intestines, kidneys, metabolism, immunity, and tumors, regulating hormone secretion, immune function, and cell proliferation and differentiation (65).

The association between vitamin D and autoimmune thyroid disease (AITD), while substantiated by clinical evidence, reveals a complex picture when examined across different levels of investigation. Clinical studies (as shown as **Table 1**) consistently demonstrate an epidemiological link: AITD patients overall have a higher prevalence of vitamin D deficiency, AITD patients had lower levels of 25(OH)D compared to controls (66–69). A significant inverse correlation is often reported between vitamin D levels and anti-thyroid antibody titers (70). However, these observational data are inherently limited by confounding factors such as seasonal variation, sun exposure, and BMI, which preclude causal inference regarding whether vitamin D deficiency is a cause or a consequence of AITD.

The mechanistic basis for vitamin D’s immunomodulatory role is well-established in the majority of studies. The nuclear vitamin D receptor (VDR) is expressed on immune cells, including dendritic cells (DCs), monocytes, and lymphocytes (71), plays a crucial role in initiating immune responses and are the most potent antigen-presenting cells (72). 1,25-(OH)_2_D_3_ regulates gene expression by binding to intracellular VDR, participating in immune regulation, forming the cellular and molecular basis for vitamin D’s involvement in the pathogenesis of AITD (65). Vitamin D enhances innate immune responses while suppressing adaptive immune responses, increasing immune tolerance, and reducing the incidence of autoimmune diseases (73). Tregs cells play a crucial role in establishing and maintaining immune tolerance and suppressing autoimmune diseases, thereby regulating the normal functioning of the immune system. Studies have shown that vitamin D enhances Treg cell function, promotes Th2 cell differentiation and maturation, and inhibits Th1 cell activation (74). First, 1,25-(OH)_2_D_3_ inhibits B cell proliferation and differentiation into plasma cells, reducing the secretion of autoantibodies targeting thyroid cells; second, it directly or indirectly promotes plasma cell apoptosis through the Th cell pathway, preventing complement activation-mediated thyroid cell death. Exogenous antigens stimulate DC activation, initiating the primary immune response. DCs recognize self-antigens to induce immune tolerance in T lymphocytes, thereby suppressing autoimmune reactions that may harm the host. 1,25-(OH)_2_D_3_ inhibits the secretion of cytokines IL-12 and IL-23, which are associated with DC differentiation and maturation, promoting T cell differentiation toward the Th2 cell phenotype. Concurrently, it enhances DC-mediated Treg cell secretion of IL-10, suppressing Th1 cell activity, inhibiting CD4+ T cell differentiation into Th17 cells, and blocking Th17 cell secretion of IL-17, thereby preventing cell-mediated cytotoxic destruction of thyroid tissue. Additionally, 1,25-(OH)_2_D_3_ blocks nuclear factor κB activation and binding to nuclear factor κB consensus sequences, downregulates IL-8 and IL-12 cytokine secretion, weakens DC antigen presentation and immune response activation functions, and reduces T cell numbers and activity in thyroid tissue (75). These effects collectively form the immune protective network of vitamin D in AITD.

Although existing evidence suggests that 1,25-(OH)_2_D_3_, as an immunomodulator, multi-pathway inhibits reactive T lymphocytes from attacking self-antigens, suppresses abnormal immune responses, reduces pro-inflammatory factor production, regulates apoptosis to protect thyroid tissue, and supports the potential value of vitamin D in the prevention and treatment of AITD, further research is needed to address the following key issues: (1) Determining the optimal dose and timing of vitamin D supplementation therapy; (2) Elucidating the synergistic mechanisms between vitamin D and other trace elements (such as selenium); (3) Exploring the differential regulatory effects of vitamin D on different AITD subtypes (such as GD and HT). Future research should focus on establishing the causal relationship between vitamin D status and clinical outcomes in AITD to provide more reliable evidence for clinical intervention.

### Zinc and AITD

Zinc serves as a cofactor for more than 300 metalloenzymes. It contributes to maintaining thyroid hormone receptor activity and modulates the function of the hypothalamic-pituitary-thyroid (HPT) axis (76–79).Zinc plays a multifaceted role in thyroid function regulation as an essential component of the thyroid antioxidant system. It serves as a key cofactor for superoxide dismutase (SOD) (80) and is involved in regulating serum T3, T4, and TSH levels (81).

Zinc is closely related to the immune system and is an essential substance for lymphocyte differentiation and maturation. It mediates cellular immune function, induces the synthesis of T lymphocytes and cytokines such as IL-1, IL-6, and TNF-α, and enhances interactions between immune cells. The association between zinc status and autoimmune thyroid disease (AITD) is supported by clinical observations (as shown as **Table 1**) but remains contentious. While some human studies report a high prevalence of zinc deficiency in patients with thyroid dysfunction and a positive correlation between serum zinc and thyroid autoantibody titers in patients with autoimmune thyroiditis (82, 83). However, these findings are challenged by a well-controlled survey showing no significant difference in mean serum zinc concentrations between women with Hashimoto’s thyroiditis and matched healthy controls (84). This discrepancy may stem from variations in study populations, thyroid dysfunction stages, or comorbidities influencing zinc status. Notably, more robust case-control evidence indicates a significantly higher incidence of hypozincemia in patients with overt hypothyroidism and hyperthyroidism compared to healthy individuals (85), reinforcing the potential link between zinc deficiency and thyroid autoimmunity. However, the exact molecular mechanisms underlying this association and the potential of zinc supplementation as a therapeutic intervention require further elucidation through well-designed longitudinal and interventional studies.

### Iron and AITD

Iron is an essential cofactor for TPO, fulfilling a dual role in thyroid hormone synthesis: it facilitates crucial redox reactions and maintains the enzyme’s catalytic activity (86). Consequently, iron deficiency impairs TPO function and thereby disrupts thyroid hormone synthesis. Additionally, iron serves as a key regulatory factor in epigenetic modification processes. Its deficiency may lead to genomic-level alterations, potentially triggering or exacerbating the onset and progression of autoimmune thyroid diseases. This underscores iron’s crucial role as a cofactor for thyroid peroxidase in hormone synthesis. Clinically, the correction of this deficiency is paramount, as timely iron supplementation is recognized as an indispensable component of managing Hashimoto’s thyroiditis (87). From a physiological perspective, substantial evidence from both animal models and human studies (as shown as **Table 1**) has established that nutritional iron deficiency impairs thyroid metabolism, consistently leading to reduced plasma T4 and T3 levels, diminished peripheral T4-to-T3 conversion, and elevated TSH (88–90). However, the association between iron deficiency and the autoimmune process itself is confounded by high rates of comorbidity. AITD patients frequently present with iron deficiency, largely attributable to coexisting autoimmune gastritis (impairing absorption) and celiac disease (causing loss) (91).This epidemiological link complicates the distinction between a mere comorbidity and a potential pathogenic driver of autoimmunity. Consequently, while the biochemical consequences of iron deficiency on thyroid hormone synthesis are well-documented, the specific immunological mechanisms by which it might initiate or exacerbate the autoimmune response in AITD remain an important area for future investigation.

### Copper and AITD

Copper (Cu), an important immune-modulating trace element, exists in plasma primarily in the form of ceruloplasmin-bound copper, accounting for over 90% of its total plasma concentration. It plays a role in both innate and adaptive immune responses (92). Research indicates that copper deficiency can lead to reduced ability of neutrophils to produce reactive oxygen species, decreased T-cell proliferation, and lowered IL-2 secretion, significantly impairing the body’s immune defense functions (92). In the context of thyroid autoimmunity, Copper influences thyroid function through a dual mechanism: it modulates the activity of enzymes critical for thyroid hormone synthesis and interacts with the thyroid hormone receptor β (TRβ) signaling pathway (93, 94). Additionally, copper’s redox properties may influence the progression of AITD by regulating oxidative stress levels within thyroid cells.

Current evidence linking copper (Cu) to autoimmune thyroid disease remains predominantly associative, inconsistent, and lacking mechanistic depth. The available evidence, consisting solely of clinical observations, presents substantial contradictions. For example, one study reported a positive correlation between serum cobalt and thyroid autoantibodies (43),whereas a later study found no significant association (95).Similarly, although the copper-to-selenium (Cu/Se) ratio has been hypothesized to influence thyroid function in hypothyroidism (96),analysis of 323 patients revealed no significant difference in serum copper levels between hypothyroidism and autoimmune thyroiditis groups (97) (as shown as **Table 1**).These discrepancies likely arise from methodological limitations, including uncontrolled confounders such as systemic inflammation—which elevates serum copper levels as an acute-phase reactant—and heterogeneity in patient populations and dietary intake. Furthermore, the complete absence of experimental data from cellular or animal models precludes determination of causal roles versus epiphenomena. Establishing pathogenic roles for copper in AITD therefore requires a transition from observational studies to hypothesis-driven experimental research examining specific mechanisms, such as modulation of oxidative stress or immune cell function in the thyroid microenvironment.

### Magnesium and AITD

Magnesium (Mg), an essential divalent cation, influences thyroid function by modulating the bioavailability and tissue distribution of selenium (98). This action indirectly supports the conversion of T4 to T3, as selenium is an essential cofactor for the deiodinase enzymes responsible for this metabolic step (92). Magnesium deficiency can lead to reduced serum selenium levels, thereby affecting the activity of selenium-dependent proteins such as GPx (98).

Although clinical correlations support an association between magnesium (Mg) status and AITD, mechanistic insights remain limited. Human studies provide evidence for this link at the clinical level. For example, serum Mg levels correlate with thyroid function parameters in TPOAb-positive subclinical Hashimoto’s thyroiditis (92). A large cross-sectional study (as shown as **Table 1**) further showed that severe hypomagnesemia (serum Mg < 0.55 mmol/L) is associated with higher TgAb positivity and increased risk of overt hypothyroidism (99). However, these epidemiological findings share a critical limitation: they cannot establish causality. The association may be confounded by dietary patterns, comorbidities, or medications influencing both Mg levels and thyroid function. Furthermore, experimental data from cellular or animal models are lacking. The molecular mechanisms of Mg in AITD-such as effects on T-regulatory cell function or oxidative stress in thyrocytes-remain speculative. Therefore, establishing causation requires integrated approaches combining mechanistic studies in cellular and animal models with prospective clinical cohorts.

## Discussion

AITD substantially impair patients’ quality of life and impose a significant socioeconomic burden, attributable to the costs associated with long-term pharmacotherapy, frequent monitoring, and complication management. Accumulating evidence indicates that early assessment and correction of trace element imbalances-particularly involving iodine, selenium, and vitamin D-may provide a cost-effective adjunct strategy to slow disease progression and improve patient outcomes. This review systematically examines the roles of multiple trace elements in AITD pathogenesis. Their imbalances-whether deficient or excessive-can disrupt thyroid immune homeostasis and act as environmental triggers. However, significant variations exist in their mechanisms of action, clinical relevance, and the depth of supporting research.

Iodine exhibits a classic dual role in AITD, characterized by a U-shaped dose-response curve. Excessive iodine not only inhibits thyroid peroxidase activity and sodium-iodide symporter expression but also compromises immune tolerance by inducing reactive oxygen species, promoting Th17 cell proliferation, and suppressing autophagy. Critical knowledge gaps persist, particularly in defining the optimal iodine intake for genetically susceptible populations and understanding its interactions with other elements, such as selenium. Future studies should establish personalized iodine targets through prospective cohorts and elucidate underlying molecular pathways using experimental models.

Selenium exerts protective effects mainly via selenoproteins, which contribute to (1) antioxidant defense through hydrogen peroxide neutralization, (2) immunomodulation by supporting regulatory T cells and suppressing Th1/Th17 responses, and (3) hormone metabolism via deiodinases. Although selenium supplementation can reduce thyroid peroxidase antibody and thyroglobulin antibody titers, randomized trials are often limited by small sample sizes, short duration, and protocol heterogeneity, precluding consensus on its long-term efficacy and optimal dosing. Selenium’s dose–response may also follow a U-shaped curve, underscoring the need to avoid excess. Future research should prioritize large, long-term, multicenter randomized trials and explore molecular mechanisms such as epigenetic regulation of T cell differentiation.

Vitamin D modulates immunity primarily via the vitamin D receptor on immune cells, inhibiting dendritic cell maturation, promoting Th2 and Treg differentiation, suppressing Th1 and Th17 pathways, and reducing autoantibody production. Cross-sectional studies consistently associate lower serum vitamin D levels with AITD and higher autoantibody titers. However, the causal direction remains unclear, and optimal serum targets, dosage, and duration of supplementation are undefined. Future work should employ prospective cohorts and interventional trials to clarify causality and differential effects in Graves’ disease versus Hashimoto’s thyroiditis.

Research on zinc, iron, copper, and magnesium in AITD remains less developed. Zinc supports antioxidant defense and hormone receptor function, yet its immunomodulatory mechanisms are poorly understood. Iron deficiency impairs thyroid peroxidase activity, but comorbid conditions often confound its association with AITD. Copper may influence oxidative stress, although reported findings are inconsistent. The copper-to-selenium ratio may be more clinically relevant than the absolute concentration of either element. Magnesium indirectly affects thyroid function via selenium bioavailability, but direct molecular insights are lacking. Research on these elements commonly suffers from limitations such as small-scale observational designs, scarce mechanistic data, and unvalidated benefits of supplementation. Future studies should identify molecular targets in the thyroid immune microenvironment and evaluate combined supplementation strategies.

In summary, trace elements form a complex regulatory network in AITD pathophysiology. Current evidence is heterogeneous, primarily focusing on the population-level effects of individual elements. Substantial gaps remain in understanding individual variability, long-term outcomes, and interactions between different elements. However, this review has some limitations, including the predominance of observational studies, heterogeneity in populations and interventions, and insufficient exploration of trace element interactions and cumulative effects.

Clinically, assessing trace element status-such as selenium, vitamin D, ferritin, and zinc-should be considered in AITD management, especially in refractory or nutritionally at-risk cases. Correcting deficiencies may serve as adjuvant therapy: for example, selenium supplementation (e.g., 100-200 μg/day) can lower TPOAb titers in Hashimoto’s patients (100, 101), and The World Health Organization (WHO) has made recommendation on the dose of selenium for adults to be 30 to 40 μg/day and stated that daily intake up to 400 μg selenium shall be considered safe (102), and vitamin D should be supplemented to achieve serum 25(OH)D > 30 ng/mL (103). Dietary recommendations should emphasize a balanced intake of specific nutrients: adequate but not excessive iodine from iodized salt; selenium from nuts and fish; and zinc and iron from sources such as lean meat, legumes, red meat, and spinach.

**Figure 1:**
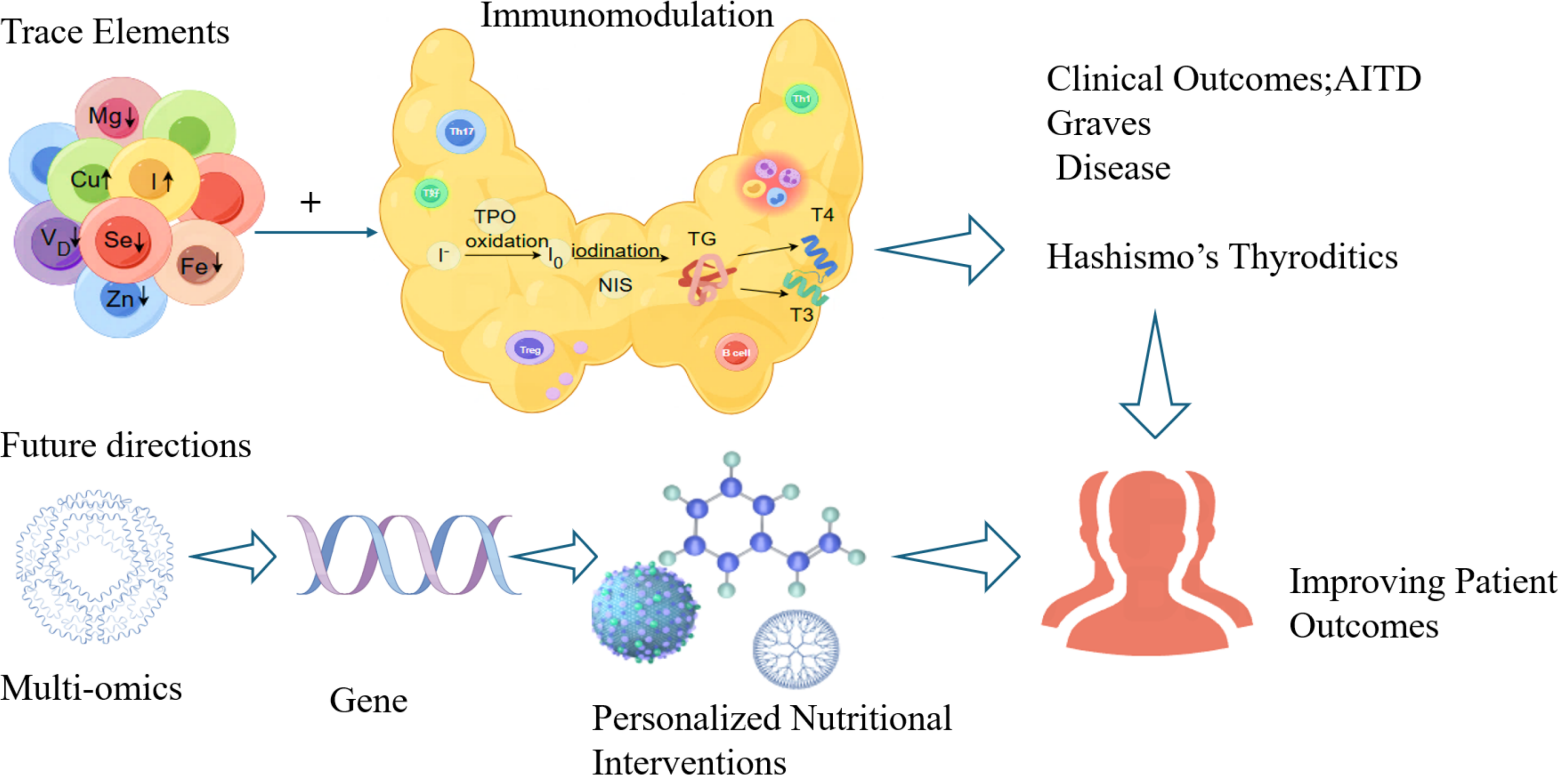
The future research directions on trace elements and AITD.

Future research should define precise reference ranges and validate supplementation efficacy through rigorous trials. Mechanistic studies ought to clarify the roles of trace elements in thyroid tissue and immune cells, particularly their regulation of key signaling pathways. Integrated multi-omics approaches-metagenomics, transcriptomics, proteomics, metabolomics and genetics-will help uncover pathogenesis mechanisms and immune-regulatory networks. Long-term prospective studies and well-designed interventions are essential to evaluate effects on thyroid function, immune response, and patient-centered outcomes. Ultimately, personalized nutritional strategies-incorporating genetic background, trace element status, and gut microbiota composition-represent a promising direction for the precise prevention and management of AITD (**Figure 1**).
